# The state of distance healthcare simulation during the COVID-19 pandemic: results of an international survey

**DOI:** 10.1186/s41077-022-00202-7

**Published:** 2022-04-05

**Authors:** C. Buléon, J. Caton, Y. S. Park, S. Eller, M. Buyck, S. Kardong-Edgren, B. M. Walsh, I. T. Gross, J. Maxworthy, G. Reedy, J. C. Palaganas

**Affiliations:** 1 Department of Anesthesiology, Intensive Care and Perioperative Medicine, Caen Normandy University Hospital, Caen, France; 2 Medical School, University of Caen Normandy, Caen, France; 3 Center for Medical Simulation, Boston, MA USA; 4 Division of Hospital Medicine, Stanford University School of Medicine, Standford, CA USA; 5 Department of Anesthesia, Critical Care, & Pain Medicine, Harvard Medical School, 36 1st Avenue, Boston, MA 02129-4557 USA; 6 Department of Immersive Learning and Learning Spaces, Stanford University School of Medicine, Standford, CA USA; 7 Department of Pediatric Emergency, Sainte-Justine Hospital University Center, Montreal, Canada; 8 MGH Institute of Health Professions, Boston, MA USA; 9 College of Health Professions, Boston, MA USA; 10 Department of Pediatric Emergency Medicine, Boston University School of Medicine, Boston, MA USA; 11 Department of Pediatrics, Yale University School of Medicine, New Heaven, CT USA; 12 School of Nursing and Health Professions, University of San Francisco, San Francisco, CA USA; 13 Faculty of Life Sciences and Medicine, King’s College London, London, UK

**Keywords:** Simulation, Education, Distance, COVID-19, Pandemics, Surveys and questionnaires, Learning, Technology

## Abstract

**Background:**

The coronavirus pandemic continues to shake the embedded structures of traditional in-person education across all learning levels and across the globe. In healthcare simulation, the pandemic tested the innovative and technological capabilities of simulation programs, educators, operations staff, and administration. This study aimed to answer the question: *What is the state of distance simulation practice in 2021?*

**Methods:**

This was an IRB-approved, 34-item open survey for any profession involved in healthcare simulation disseminated widely and internationally in seven languages from January 14, 2021, to March 3, 2021. Development followed a multistep process of expert design, testing, piloting, translation, and recruitment. The survey asked questions to understand: *Who was using distance simulation? What driving factors motivated programs to initiate distance sim? For what purposes was distance sim being used? What specific types or modalities of distance simulation were occurring? How was it being used (i.e., modalities, blending of technology and resources and location)? How did the early part of the pandemic differ from the latter half of 2020 and early 2021? What information would best support future distance simulation education?* Data were cleaned, compiled, and analyzed for dichotomized responses, reporting frequencies, proportions, as well as a comparison of response proportions.

**Results:**

From 32 countries, 618 respondents were included in the analysis. The findings included insights into the prevalence of distance simulation before, during, and after the pandemic; drivers for using distance simulation; methods and modalities of distance simulation; and staff training. The majority of respondents (70%) reported that their simulation center was conducting distance simulation. Significantly more respondents indicated long-term plans for maintaining a hybrid format (82%), relative to going back to in-person simulation (11%, *p* < 0.001).

**Conclusion:**

This study gives a perspective into the rapid adaptation of the healthcare simulation community towards distance teaching and learning in reaction to a radical and quick change in education conditions and environment caused by COVID-19, as well as future directions to pursue understanding and support of distance simulation.

**Supplementary Information:**

The online version contains supplementary material available at 10.1186/s41077-022-00202-7.

## Background

### The problem

COVID-19 forced a predicted decade’s worth of digital platform adoption to occur in a matter of months [1]. Digital and distance technology for education and healthcare had been adopted unevenly up to that point, especially in community and rural hospitals, which tended to lag behind the developments of larger urban tertiary, quaternary, and teaching hospitals. The simulation and technology adoption which has occurred during the pandemic is anticipated to change education permanently [2], and simulation-based education will be changed as well [2]. Furthermore, we need to ensure the education of providers to continue safe care for patients during the global pandemic: healthcare is one of the very few industries that cannot pause during a pandemic [3].

As the simulation community embraced distance approaches during the pandemic, members of a number of simulation societies came together in a collaborative approach as “The Healthcare Distance Simulation Collaboration.” This collaboration quickly created groups to explore the multiple facets and potential resources of distance simulation: (1) a taxonomy group looking at definitions and terminology used in distance simulation, (2) a group to perform a scoping review of distance simulation in peer-reviewed and grey literature, (3) a summit-planning group to bring all teams together to share and disseminate findings, (4) a pictogram development group exploring ways to express the concept without words since consensus on terminology was seemingly difficult and article texts seemed more confusing to the methodology than the figures embedded, and (5) a team working on future research questions in distance simulation. As the scoping review team completed their review of the literature, the question arose: What about those simulation programs that are too busy actually doing this work to publish their approaches? How do we capture the breadth, the scale, and the creativity of the simulation education community as it seeks to continue its work in spite of pandemic restrictions?

### Addressing the problem

To understand the full picture, we expanded the scoping review into this additional separate study: a large-scale international survey to attempt to capture what types of online, remote, and distance simulation methods and approaches are being used. A consortium of members representing international medical and health profession societies have formed together in this large-scale survey project, including presidents of simulation societies, editors-in-chief of simulation journals, medical and nurse simulation leaders and researchers. The shared intention was that this study provides insight into both the actual state of simulation practice, as well as the creative and survival spirit of the field, showing how simulation has striven to meet current and future needs.

### Aims and objectives

The survey sought to explore current innovative methods used for and in distance simulation in response to COVID-19. The aim of the work was to (1) provide a snapshot of current methods used to inform future development, support, and research; (2) assist in defining distance simulation and its characteristics, to develop an understanding of terminology for ease of standardization and future resource searches; and (3) provide a knowledge and a resource base for simulationists and health professions educators to inform developing and future standards of best practice. This study, therefore, asked the following question: *What was the state of distance simulation practice in 2021?* For the purposes of the study, distance simulation could include any number of different modalities, such as remote simulation, telesimulation, and virtual simulation. In this context, the research specifically sought to explore:

- Who was using distance simulation and how did this change over time?
- What driving factors motivated programs to initiate distance simulation and for what purposes were programs using it?
- For what purposes was distance simulation being used?
- What specific types or modalities of distance simulation were occurring and how were they being implemented?
- How were staff recruited and trained to create and facilitate distance simulation?
- How did distance simulation compare with in-person simulation in terms of specific challenges and satisfaction for each stakeholder group?
- What information would best support future distance simulation education?

## Methods

### Survey design

This open survey was designed to be completed by adults who have allocated or dedicated time working in healthcare simulation. This included administrators, educators, or clinical providers at any level of competency and with any amount of clinical or simulation experience. The design and results of this survey are reported here based on the guidelines for reporting results of internet e-surveys (CHERRIES) [4].

### Development and testing

The creation of the survey followed several steps [5]. The survey was developed by a core group of eight experts in healthcare simulation research and practice (CB, MB, SE, SKE, JM, BW, GR, JP), identified by their leading roles within the healthcare simulation community. The group included two experts in survey design (JC, YSP). The survey design emerged from discussion among the research team regarding potential characteristics of distance simulation which could help to answer the research question, along with aspects of distance simulation as reflected in the scoping review of distance simulation [6]. Questions were constructed and reviewed by a steering group (13 experts in healthcare simulation research and practice), drawing on their experience as simulation practitioners and researchers, refined iteratively, and checked against existing frameworks such as the healthcare simulation research reporting guidelines [7]. The draft survey was created and circulated to the research team and larger collaboration for validation and review, and items continued to be iteratively refined. The survey was piloted (before ethical review) by similar subjects of the targeted population recruited by the experts among their network, and the final wording of some items was refined based on the pilot feedback. Overall, the survey went through five major revisions prior to being finalized (Additional file 1). The survey was translated to be available in seven languages: English, French, German, Italian, Korean, Mandarin, and Spanish. Each translation was completed by a member of the broader research team who was a proficient user of the target language, and thus all translators had expertise in medical simulation. During pilot testing the survey was piloted in all languages, and feedback from pilot testers was then incorporated into the final version of the survey in each language.

### Institutional Review Board approval and informed consent process

The survey was reviewed by the Mass General Brigham Human Research Committee on January 13, 2021 (ID 2020P003981, chair O. Johnson-Akeju) and was determined exempt from further review. In accordance with ethical research practice, participants were given information about the scope and aims of the research, the length of the survey (approximately 15 min), the research team conducting the survey, and data confidentiality, as well as the scope of their own participation and their rights to withdraw their participation, and were asked to confirm their understanding before beginning the survey. Completion of the survey thus implied that participants had read and understood this information and had consented to participate in the research. Participation was anonymous, in that participants were not asked for their name or other personal identifying characteristics. Participants were asked to provide voluntary information on their institutional affiliation, to help facilitate sorting responses from varying simulation centers, but this question was optional. The survey was hosted by, and the raw data was stored in, the Qualtrics (Qualtrics™, Provo, UT, USA) online platform. The data were accessible only to the researcher who programmed the survey (JC), who downloaded the unidentifiable anonymous results for analysis. If any identifiable data was provided in free-text qualitative responses, this was redacted prior to analysis.

### Recruitment process

Using existing professional and online social networks, the survey was distributed widely as an “open survey” within the healthcare simulation community. The survey link was distributed over the internet where anyone with the link could participate. The survey was advertised via social media and email distribution lists from local, regional, national, and international simulation centers and organizations (Appendix 1). Reminders were posted on social media and via the email distribution lists used to disseminate the survey. The team reviewed geographic responses in weekly meetings and sought to target countries with fewer responses by asking simulation leaders residing in those regions to share via social media.

### Survey administration

The survey was a web-based survey administered using Qualtrics software (Qualtrics™, Provo, UT). Due to the descriptive nature of the survey, there was no randomization of the questions. Adaptive questioning was used in the survey design to ensure that respondents answered questions relevant to their experience when possible. The questionnaire had a total of 34 questions, with two to eight questions displayed per page. A completeness check was performed before the survey was distributed. All questions on the survey were optional, except the first two questions, which were mandatory (“*Did your simulation center conduct distance simulation activities prior to the COVID-19 pandemic?*” and “*Does your simulation center currently conduct distance simulation activities?”*). If a respondent answered “no” to both mandatory questions, the survey logic skipped to a short demographics section. Any “yes” answers continued to the full survey. All items provided a non-response option, such as “*not applicable*” or “*not sure*,” to ensure that all participants could select an option that matched their knowledge or experience. A back button was included in the survey design to allow respondents to review and change their answers throughout the survey. Participation was voluntary, no incentives were provided, and respondents could choose to exit the survey at any time. The survey link was open for responses over a seven-week period from January 14, 2021, to March 3, 2021.

### Drop rate and duplicate entries

Respondents were prevented from taking the survey more than once using a functionality in the Qualtrics platform, which uses cookies to prevent users from accessing the survey link more than once. IP addresses were not collected as part of the dataset. The drop rate was calculated by dividing the number of respondents who completed less than 5% of the survey by the total number of respondents who started the survey by clicking past the informed consent page.

### Data management

Data were cleaned, compiled, and analyzed using Stata 16 (College Station, TX).

#### Data cleaning and compilation

To account for incomplete responses and respondent attrition while completing the survey, we dropped records with less than 5% of other answered questions. It accounted for about 12.7% of total responses removed from the compiled data. Data were also verified for consistency, checking for straight-lining errors (respondent checking the same response option throughout the survey) and examining data quality for skip patterns and lagged responses (e.g., survey responses lasting over one day). Responses to survey items were dichotomized to facilitate analysis and interpretation, combining the top two and the bottom three response options in the five-point ordinal scale. Because we used an open survey design where anyone with access to the link could respond to the survey, we could not calculate a response rate.

#### Data analysis

We calculated descriptive statistics for the dichotomized responses, reporting frequencies and proportions. In addition, we compared response proportions with (1) status in implementing distance simulation (continuing or new distance simulation) and with (2) respondent role in the simulation center (administrative/leadership role versus others). Responses were treated as independent records, making the assumption that each respondent represented different simulation programs or centers, allowing broader inferences with data collected. Comparison in proportions was conducted using chi-squared tests. We also used logistic regression to estimate odds ratios for factors predicting outcomes. For the questions that had optional short free-text, the responses were analyzed by word frequency (e.g., for “which platform did you use?”; had word counts for “Zoom”, “Teams”, and other software names; for “other profession” each profession listed was analyzed by frequency combining professions listed under different names, i.e., different languages).

## Results

We analyzed and organized the survey results to answer the research questions outlined in the “Aims and objectives” section. In the following paragraphs, we will describe who reported using distance simulation, as well as how this changed over time: from before the pandemic, to the beginning of the pandemic, and then to the latter part of 2020. We will then examine the driving factors motivating programs to initiate distance simulation, and the main reported purposes for distance simulation. Next, we explore the modalities of distance simulation being used, and how programs reported they were implementing these modalities. Then, we will present results related to personnel training and recruitment for distance simulation. Finally, we will report survey data on the specific challenges of distance simulation relative to in-person simulation and the perceived satisfaction of various stakeholders with distance simulation.

### Sample demographics

We gathered data from 708 respondents. Following data cleaning and compilation, 618 responses were included in this analysis. The median time to complete the survey was 9 min 17 s, with an interquartile range of 2 min 23 s to 16 min 48 s.

The respondents came from 32 countries and within the USA from 44 states. Forty-one percent of respondents dedicated more than 75% of their time to simulation, 16% dedicated 50–75% of their time to simulation, and 43% dedicated less than 50% of their time to simulation. The survey allowed respondents to select multiple applicable roles: 65% described themselves as simulation educators, 42% as simulation program directors and/or administrators, 21% as simulation researchers, 14% as simulation operation specialists or technicians, and 7% as standardized patient program directors or administrators.

### Prevalence of distance simulation

The majority of respondents (70%) reported that their simulation center was conducting distance simulation at the time they completed the survey and, of those, 28% of respondents reported that their simulation center conducted distance simulation prior to the COVID-19 pandemic, representing a 42% increase in transition to distance simulation (*p* < .001). Among the respondents currently conducting distance simulation, 60% did not previously use distance simulation. Among respondents who used distance simulation prior to the COVID-19 pandemic, 7% were no longer using distance simulation at the time of the survey.

**Fig. 1:**
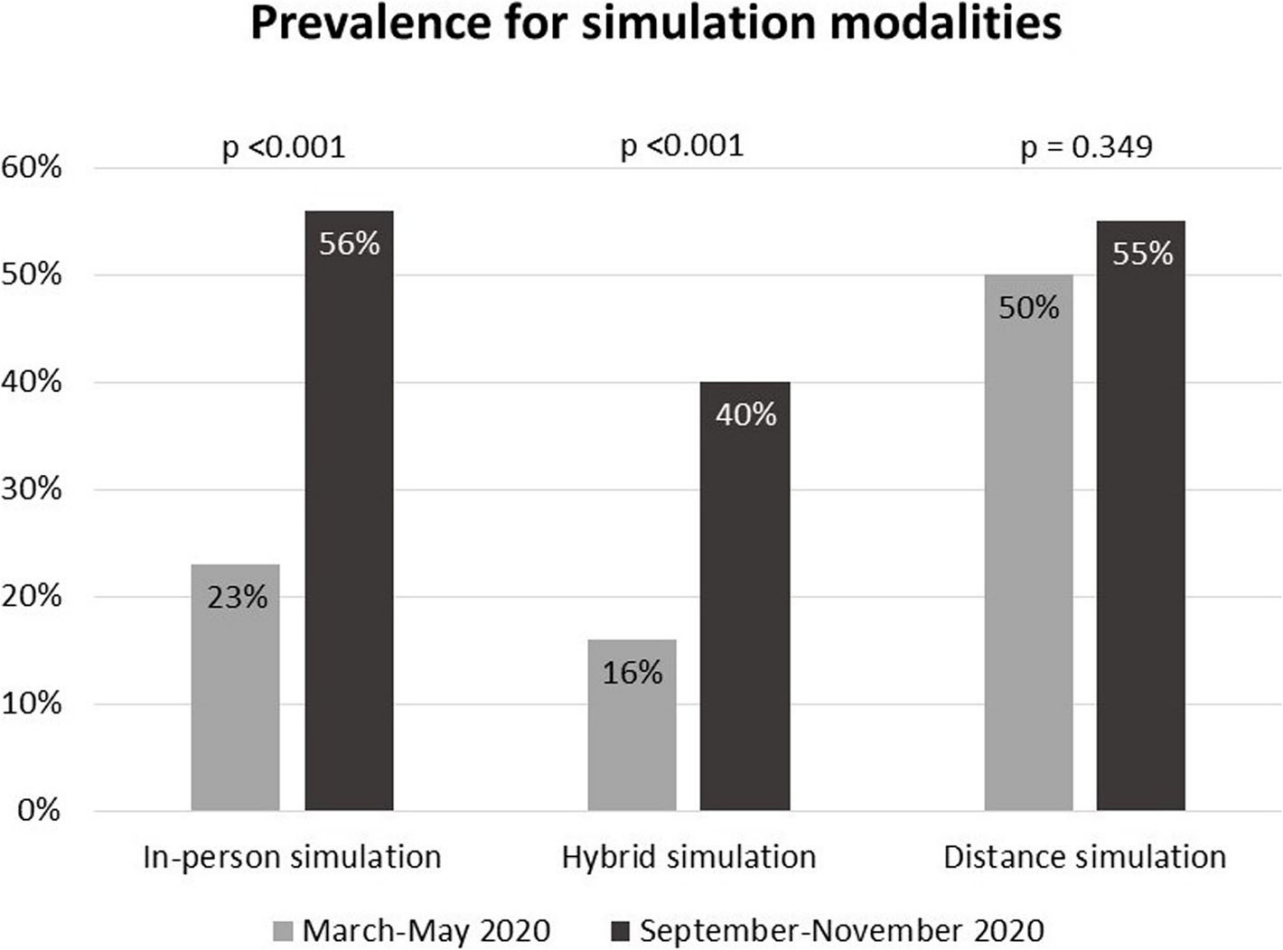
Prevalence for simulation modalities during first COVID-19 wave and following academic period

Distance simulation was consistently listed as the main simulation modality during 2020 (Fig. 1). Significantly more respondents indicated long-term plans for maintaining a hybrid format (82%), relative to going back to in-person simulation (11%, *p* < .001).

### Drivers and purposes for using distance simulation

We sought to understand what kinds of things respondents identified that drove the rapid engagement with distance simulation and for what purposes distance simulation was being used. There were five basic categories articulated by respondents: (1) local, regional, or national public health regulations (e.g., lockdown, reduced capacity, social distancing requirements), (2) the necessity of clinical practice time for health professions trainees (with the concomitant lack of access to patient settings due to COVID-19), (3) requirements to continue training for students or trainees, (4) the need to urgently train clinical personnel for COVID-19 response, and (5) safety concerns about ability or appropriateness of bringing together clinicians in in-person environment during the pandemic.

### Methods and modalities of distance simulation

**Fig. 2:**
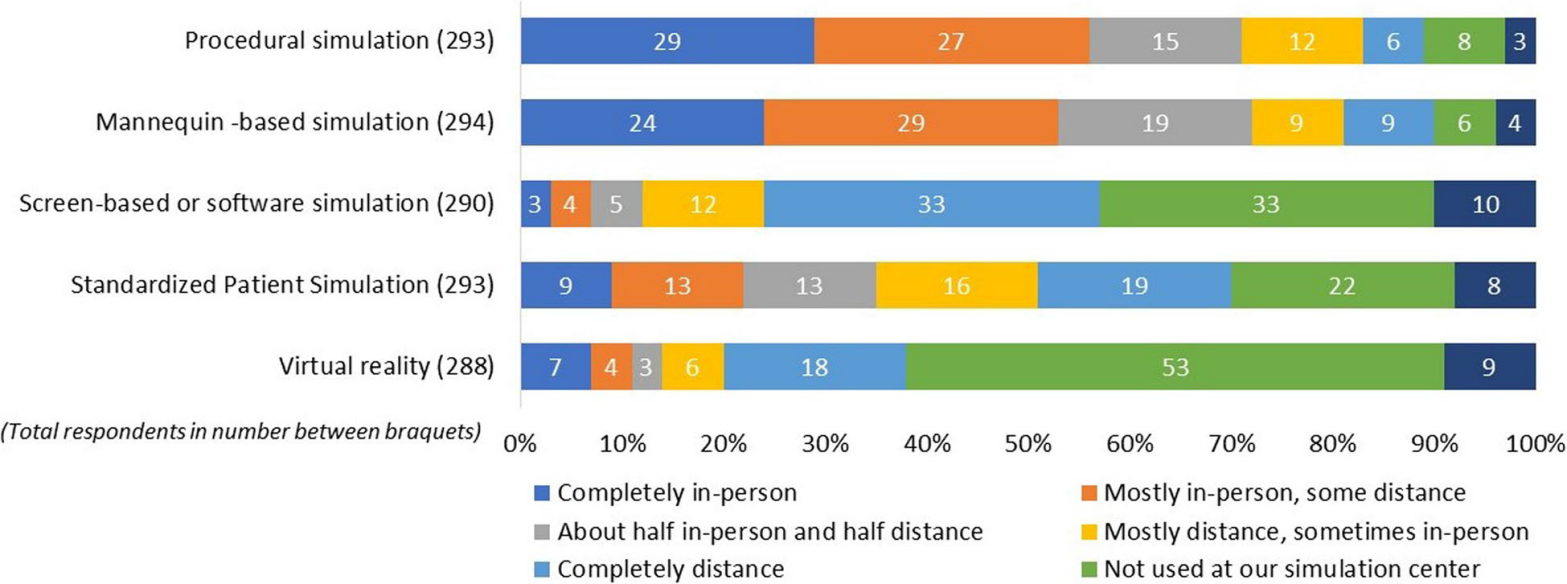
Modalities used for simulation during the COVID-19 pandemic

The type of simulation that respondents used seemed to influence the degree of shift from in-person to distance approaches, with some types of simulation more amenable to the shift than others. Overall, simulation with standardized patients was more commonly conducted as distance simulation, compared with procedural simulation or mannequin-based simulation, which was more likely to be conducted in-person (Fig. 2). Fifty-six percent of respondents continued to conduct procedural training completely or mostly in-person during the pandemic. Fifty-three percent conducted mannequin-based simulation completely or mostly in-person. On the other hand, of the centers that conducted simulation with standardized patients, 69% of respondents reported that half or more of sessions were conducted as distance simulation. The majority (57%) of respondents used commercially produced screen-based simulations, while 33% reported not using these products. More than half of respondents (53%) reported using no virtual reality simulation during the pandemic (Fig. 2).

Looking at a more granular level within each simulation type, we found differences based on participant role in the proportion of participants that were in-person versus distanced. These findings are described in detail in Table 1. For simulation focused on procedural skills, respondents reported that the majority of participants (74% active learners, 74% instructors, 85% simulation techs, and 70% of administrative staff) were in-person, though sometimes separated from other participants (27% of active learners, 24% of instructors, 36% of simulation techs, and 34% of administrative staff). However, observing learners were split between in-person not separated (25%), in-person separated (30%), and distanced (44%). The breakdown was similar for clinical event-based or team-based simulation, with the majority of all participant types present in-person except for observing learners, who were split between in-person (51%) and distanced (49%) (Table 1).

**Table 1:** Participants’ location and synchronicity according to simulation modality (in-person or distance) and simulation type (data in percentage)

Simulation type	Participants	In-person		Distance simulation		
		Not separated	Separated	Synchronous with Interaction	Synchronous without Interaction	Asynchronous
Procedural simulation	Active learners	47	27	21	3	2
	Observing learners	25	30	31	6	7
	Instructors	50	24	22	2	2
	Simulation techs	49	36	11	2	3
	Administrative staff	36	34	18	3	9
Clinical event-based or team-based simulation	Active learners	45	18	33	2	2
	Observing learners	23	28	41	5	3
	Instructors	41	25	30	2	2
	Simulation techs	43	37	15	3	3
	Administrative staff	34	34	18	5	9
Simulation with simulated patients	Active learners	26	11	56	4	2
	Observing learners	16	18	55	9	2
	Instructors	28	17	46	4	5
	Simulation techs	31	27	34	7	1
	Administrative staff	29	25	32	8	6
	Simulated patients	26	15	53	3	3

In contrast, for simulation with simulated patients, the majority of learners (active (62%) and observing (66%)), instructors (55%), and simulated patients (59%) were reported as participating in distance simulation, whereas for this type of simulation, simulation techs (58%) and administrative staff (54%) were still reported as in-person by the majority of respondents (Table 1).

Additionally, when looking at responses pertaining to distance simulation specifically, respondents reported that distance simulation mostly occurred as synchronous sessions with interaction between participants, as opposed to synchronous sessions without interaction or asynchronous sessions. This finding was true regardless of simulation type or participant role (Table 1).

### Staff training and recruitment

Respondents were asked about whether they received specific training to engage with distance simulation, and if so, how much and what type of training they received. A limited number of educators (39%) reported having specific training for developing distance simulation sessions. Nearly half of educators (47%) reported having some training prior to teaching in distance simulation: this consisted primarily of training in technical skills (29%), teaching and learning considerations (27%), and applications or online platforms for distance education (24%). Similarly, a limited number of respondents’ technicians (31%) reported having specific training for developing distance simulation sessions. Their specific training consisted mainly in technical skills (18%), applications or online platforms (19%), and teaching and learning considerations for distance education (11%). The text-based responses for the item, “What topics does this simulation training cover?” included--in order of frequency starting with highest: Zoom or Learning Space, coaching, telepresence robots, screen-based simulations, and power failure.

Respondents reported choosing faculty or instructors by many different means: some of the instructors volunteered for distance simulation (18%), while others were chosen by leadership (22%) or had to convert because of their assignment as faculty (32%).

### Challenges and satisfaction

**Fig. 3:**
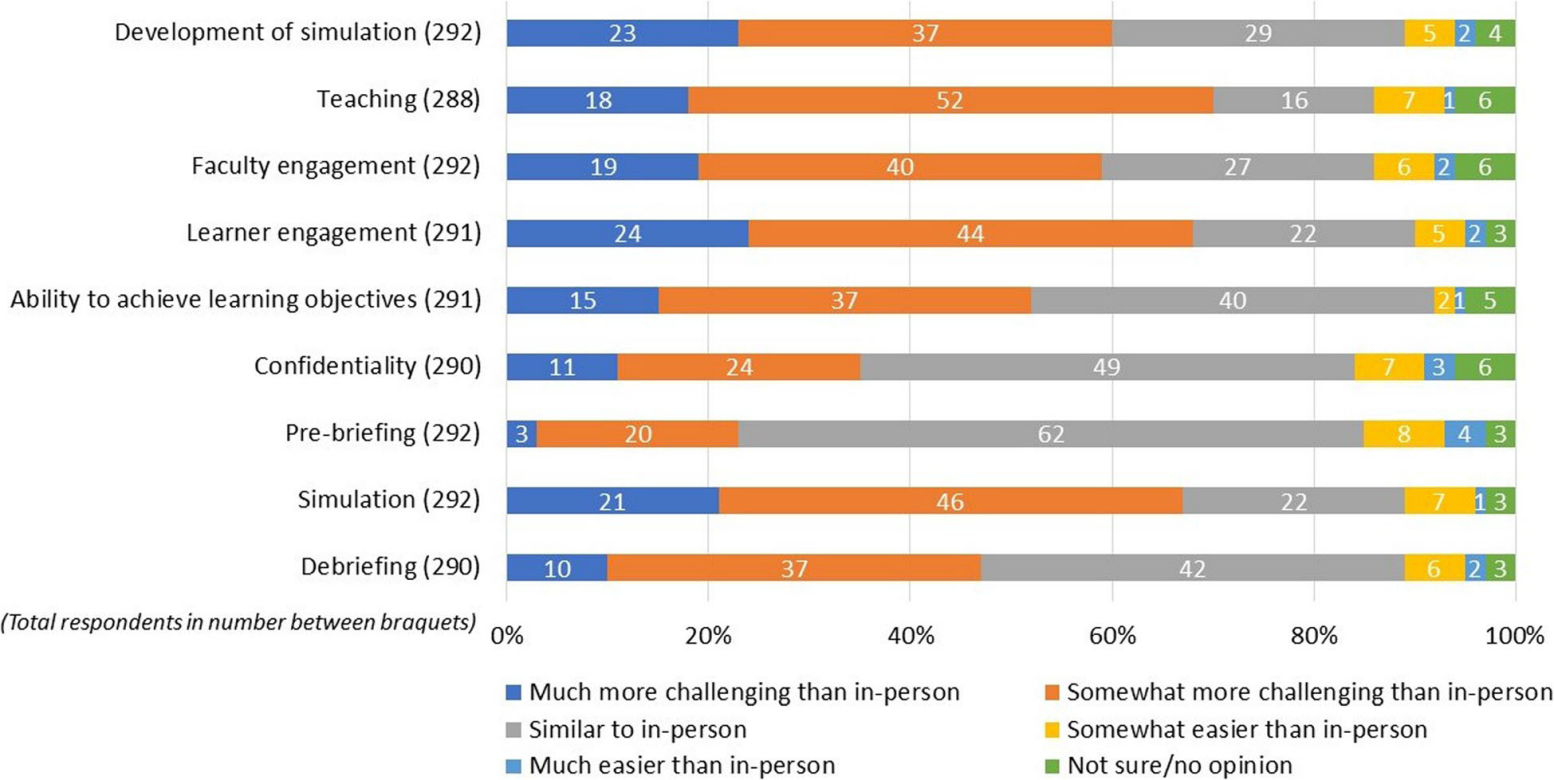
Challenge or ease of distance simulation aspects compared to in-person

Respondents were asked about how challenging they found it to participate in and administer distance simulation, as compared with in-person simulation, both in general and for specific components of simulation sessions. Most respondents reported that distance simulation was challenging for educators, compared to in-person simulation (Fig. 3). Sixty percent of respondents suggested that it was more challenging to develop distance simulation sessions; 70% found it more challenging to teach in distance simulation; and 59% found that faculty engagement in distance simulation was more challenging. Sixty-seven percent of respondents thought that learner engagement in distance simulation was more challenging than in in-person simulation. Notably, 52% of respondents suggested that the ability to achieve learning objectives was more challenging in distance simulation; however, 40% thought that it was similar to in-person simulation.

In terms of the challenges of the specific components of distance simulation sessions, respondents were asked about their experience with carrying out confidentiality, prebriefing, and debriefing when compared with in-person simulation. Fifty-nine percent of respondents reported that confidentiality was similar to or easier to carry out in distance simulation (Fig. 3). Seventy-four percent thought that prebriefing was similar to or somewhat easier to carry out than in-person simulation. While only 22% of respondents thought the challenges were similar for the simulation itself, 67% thought that distance simulation made running the simulation either much more challenging or somewhat more challenging than in-person simulation. Respondents were almost evenly split on how easy or hard debriefing was in distance simulation, with 50% reporting that debriefing was similar to or much easier than in-person simulation. Forty-seven percent reported that it was somewhat or much more challenging to debrief in distance simulation.

**Fig. 4:**
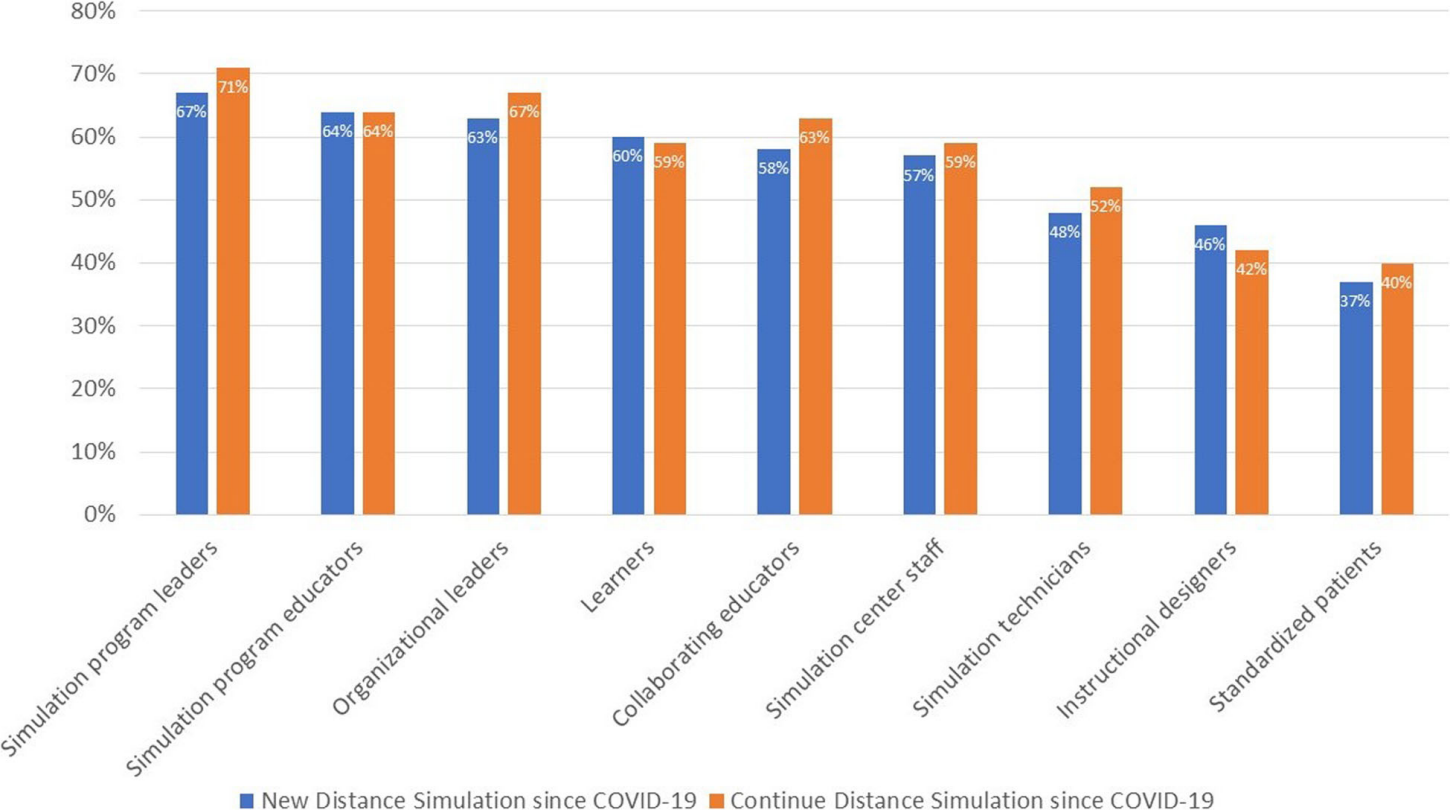
Perceived satisfaction in modality use: new versus continue distance simulation since COVID-19. Responses were dichotomized from the 5-point scale, collapsing top two categories and the bottom two categories. Percentages correspond to the rating “Quite satisfied” and “Extremely satisfied.” No significant difference exists between new and continue distance simulation since COVID-19

In light of these challenges, as well as the new opportunities that distance simulation might afford, respondents were asked about their overall satisfaction with distance simulation. Among simulation center participants, educators, and stakeholders, perceived satisfaction varied. Simulation program leaders, program educators, and organizational leaders expressed highest levels of perceived satisfaction (all > 60%). However, simulation technicians, instructional designers, and standardized patients expressed the lowest levels of satisfaction (Fig. 4).

## Discussion

Online and distance simulation increased significantly in both prevalence and diversity of modalities during the COVID-19 pandemic. The in-practice experiences of a large international group of simulation educators, staff, and researchers was reinforced by evidence from this worldwide survey on online and distance simulation practices. The simulation community embraced online and distance approaches out of a combination of pragmatism and necessity and used the opportunity to push the boundaries of distance learning in service of immersive and meaningful learning in the health professions. Some specific findings related to our research aims are notable from our analysis of the data.

### Simulation workforce satisfaction

Although the simulation community embraced distance simulation during the pandemic, doing so was not without challenges. Respondents who indicated their role as simulation center directors had the highest reported level of satisfaction with distance simulation; those in other roles (simulated patients, instructional designers, simulation technicians) reported lower levels of satisfaction. This may reflect a difference in perspective between those in different roles in simulation centers. For instance, while distance simulation could be seen to *solve* problems for simulation center directors (as discussed previously), those in other roles must do the difficult work of designing, producing, and creating meaningful immersive simulated learning environments at a distance. The mismatch in satisfaction perhaps implicates a mismatch in understanding of the work needed and the challenges encountered in order to successfully implement distance simulation. Our findings in modalities and approaches hint at this implication.

### Modalities and approaches

Simulation modalities used during the COVID-19 pandemic have been diverse. While procedural simulations and team-based simulations are mainly continuing in-person, as expected, simulations with simulated and standardized patients seem to have shifted toward online participation. This difference might be explained by the “hands-on” objectives (which necessitate in-person simulation) versus “communication” objectives (for which distance simulation is feasible) [8]. While a variety of modalities and combinations of modalities has always been both an advantage and challenge for simulationists who primarily teach in-person, the addition of a plethora of distance simulation modalities—existing and new—fundamentally and significantly expands the creative simulation space. The abundance of modalities and approaches may quickly outdate existing knowledge and requires more expansive simulation faculty education, as well as the need for simulationists to consistently stay up-to-date in simulation innovations and research.

The location of participants varied according to the modality of the simulation and seems to follow the same pattern: active learners and instructors are mainly located at the simulation center for procedural and team-based simulations, but remote for simulation with simulated and standardized patients. Literature published during the 2019 pandemic has shed some light on the physical nature and satisfaction of remote learning environments [9–11]. Continued comparative study of distance simulation learning outcomes and the human factors that affect learning as it relates to location will be needed to best inform distance simulation practices.

One important finding from this survey is the experience with the preparation and development of distance simulation as compared with in-person simulation. Overall, all the educational aspects of distance simulation were more difficult to prepare and to lead, including creation of the educational content and teaching during the simulation session. This may be because of the change of settings, the need to adapt the learning objectives for online or distance teaching, the alteration of interaction between active learners and instructors, or because of technical difficulties [12]. Training of educators and guidelines for the emerging and transforming practice of distance simulation are needed for this transition to distance simulation [11, 13–15].

### Training for simulation staff and faculty

Whether a newly developed program or one established at the start of the COVID-19 pandemic, access to technology and human resources were reported as essential elements that fostered distance simulation. The knowledge of the necessary resources is crucial to disseminate so as to promote the development and improvement of distance simulation. This, with the support of instructors, the broader organization, and the program, can convince learners and undecided staff to move forward with participation. We have a better understanding of how resources are important to implement distance simulation. Some of this came from troubleshooting in the moment and learning in real time, which while not ideal, was necessary [8]. The online environment presents new challenges that require new resources, human expertise, and needs assessments [14–17]. If such resources, whether technical and/or human, are lacking, this presents a barrier that may prevent or slow distance simulation from being further utilized in a time of great need.

Respondents reported choosing instructors by many different means: some of the instructors volunteered for distance simulation, while others were chosen by leadership or had to convert because of their assignment as faculty. Uncertainty, lack of preparation and inexperience may be daunting to instructors. Some training could help in facilitating and reassuring staff that their existing skills are valuable even in what can seem to be the very different world of distance simulation. Even if results are better in pre-existing distance simulation programs, educators (and simulation technicians) were small in numbers and thus did not undergo specific training in regards to facilitating distance simulation (less than 50%). Furthermore, of those who had been previously instructing in simulations, prior to using distance simulation, we learned that less than 50% of those had formal training. We might have anticipated that the training would have been more developed in new distance simulation programs (e.g., those who began during the pandemic period), but that was not the case. This is truly paramount if we want our programs to be well received by the learners as well as impactful.

A lack of training may be cause for discomfort for instructors and simulation technicians alike and could contribute to poor learner experience. Formal training for simulation staff may improve distance simulation quality and the experience for both learners as well as simulation staff and faculty. Among respondents who did report providing training, detailed training content was equally balanced between two major axes: (1) theoretical teaching and learning considerations and (2) technical skills and online platform (e.g., Zoom) training for instructors. For simulation technicians, this training was more focused on the technical side. Those results give us a first glance at the when, why, who, and what for the training of staff for—and ideally in the future, prior to—distance simulation.

### Limitations to the study

As in any study, there are some limitations of this work. This survey is necessarily a snapshot of the simulation community during the specific time periods queried by the survey and at the time the survey was completed (January to March, 2021). The landscape of online and distance simulation changes on a regular basis, both in response to local public health situations (as related to the ongoing pandemic) and, from the perspective of simulation educators, as new creative combinations or innovations continue to be developed, created, and realized.

There remains a challenge associated with ensuring fulsome international representation in any survey, especially as most of the members of the research group are based in, and have professional networks aligned with Europe and North America. Although the survey received responses from many countries around the world, most of the responses were from North America. The publication of the survey and its propagation in English may be a factor contributing to this, because although we were able to translate and make the survey available in a number of languages, the ability to report activity and market in a native language may still be a barrier for many.

Some other aspects of the survey design may have limited the impact of the results. For instance, the open survey design makes it challenging to estimate accurate response rate, because the denominator of the population that had access to the survey remains unknown. However, administering the survey in this way allowed us to reach respondents that may not otherwise have had access to the survey. The survey was written with perspectives regarding the simulation center (e.g., What professions does your simulation center support?) and specific types of simulations (Currently, where are the following participants located for the majority of procedural simulation at your simulation center?). Survey data were analyzed at the respondent level, generalizing responses to reflect independent program- or center-level inferences. Given the anonymity of survey responses, coupled with the voluntary nature of identifying institutional affiliation, disaggregating multiple responses possibly from the same institution was challenging to verify nor compile at the time of analysis. As such, future studies may be designed to better reflect the complexity of individual- and program-level data as part of additional data collection for more nuanced analysis. There is a possibility that the time commitment required to complete the survey (approximately 15 min) may have led to systematically fewer responses from members of the simulation community who are busy with simulation or clinical work. Indeed, 12.5% of respondents opened the survey but did not complete enough for the results to be meaningfully interpreted or included in the analysis (e.g., completed < 5%). Further, in order to protect respondents’ privacy, all but the first two questions on the survey were optional, and therefore some respondents did not answer all of the questions; this is particularly relevant for demographic questions and could have led to some item non-response error. For example, we often could not determine how many respondents came from an individual institution. This prevented potential additional analysis that requires unique associations (e.g., geographic comparisons).

### Future directions

The snapshot that was captured in this study indicates the need for a future agenda of research and further innovation in distance simulation. Respondents were clear that distance simulation is not going to disappear in a post-pandemic world. In particular, several questions emerged in our discussion of the findings. These included:

- What faculty and staff competencies would support broader implementation of distance simulation?
- What are the theoretical underpinnings of distance simulation?
- What are the best uses of distance simulation, and further, what are different modalities of distance simulation suitable for?
- What is the mix of learning goals more suitable for in-person simulation (e.g., procedural task simulation) versus other focused goals more feasible for distance simulation?
- What might emerge as best practices in distance simulation facilitation and debriefing?
- What outcome measurements may be most useful researching distance simulation?
- What measures and resources are needed?
- How would distance simulation apply in under-resourced regions?

Our committee has planned a series of efforts leading up to and including the 2023 Research Summit, culminating in a framework for future directions and research in distance simulation.

## Conclusions

This study gives a perspective into the rapid adaptation of the healthcare simulation community towards distance teaching and learning in reaction to a radical and quick change in education conditions and environment caused by COVID-19. This snapshot captures “how, when, why, who, and what” for the challenges presented by the implementation and adaptation from in-person to distance simulation during the pandemic. This understanding can help institutions, programs directors, educators, and even learners to better and more consciously draw the next steps for their simulation programs. To pursue understanding and help this transformation, future research will focus on (i) exploring how and what kinds of distance simulation are being used and (ii) understanding more about aspects of quality in distance simulation. Studying the quality of outcomes in distance simulation research can inform the development of guidelines for distance simulation education in healthcare.

## Supplementary Information

**Additional file 1.** Supplemental digital content: survey questionnaire text. Appendix 1: Social media and email advertisement for the survey.

## Data Availability

The datasets analyzed during the current study are available from the author Julia Caton on reasonable request.
