# Supplementary material for: The state of distance healthcare simulation during the COVID-19 pandemic: results of an international survey
Source: Adv Simul (Lond). 2022 Apr 5;7:10. doi: 10.1186/s41077-022-00202-7 (PMC8980782; doi:10.1186/s41077-022-00202-7)
Supplement: Supplementary file 1 — Additional file 1. Supplemental digital content: survey questionnaire text. Appendix 1: Social media and email advertisement for the survey. [file 41077_2022_202_MOESM1_ESM.docx]

Supplemental Digital Content: Survey questionnaire text.

Understanding Distance Simulation Today

We would like to invite you to participate in this 20-minute online survey. *Ethical Clearance Reference Number: Exemption 45 CFR 46.101(b)(#),*Mass General Brigham 2020P003981     What is the purpose of the study? The aim of this program of research is to understand distance simulations, inform simulationists how distance simulation is being utilized, and explore the quality of online education. We will be sending a total of 3 surveys.    Survey 1 (this survey) is to get a snapshot (environmental scan) of the landscape of distance simulation* and to explore how distance simulation is being used and implemented for various learners and to various ends. We are looking to understand: Who is using it? What motivates initiation of distance simulation? What purposes is it being used? What is being done and how is it being used? What information would best support future distance simulation?   *The term “**distance simulation**” is used to describe different modalities of simulation that can be remote simulation, telesimulation or virtual simulation. Based on Survey 1’s findings, we anticipate that Survey 2 (scheduled to be sent in April 2021) will help inform: How can educators best develop/implement distance simulation? Our goal for Survey 3 (scheduled to be sent in July 2021) is to help inform: What are the best methods for evaluations in distance simulation? Does distance simulation meet the same learning outcomes as they would in-person?   Am I the right person to complete this survey? You are eligible for this study if you are over the age of 18 and have allocated or dedicated time working in a Simulation Program. This may include administrators, educators, clinical providers at any level of competency and with any amount of clinical and/or simulation experience. Participation is completely voluntary. You should only take part if you want to, and choosing not to take part will not disadvantage you in any way. Advantages include contribution to knowledge and you will directly receive the report generated from the survey analysis.   What can I expect? If you agree to take part in this study, you will complete a survey. The survey will ask you questions about distance simulations. The survey will take you approximately 20 minutes to complete depending on how much detail you choose to give.  At the end of the survey, you will be asked if we can send you Survey 2 and Survey 3 in the future (approximately 3 to 6 months apart), and if so, to provide your email. Other than this, we will not ask you for any personally identifiable or sensitive data. You are free to withdraw at any point during the survey, without having to give a reason. Withdrawing from the study will not affect you in any way. The data submitted thus far will be collected. Once you submit the survey, it will no longer be possible to withdraw from the study because we do not collect any information about you that allows us to find and delete your response among the many responses we receive. Please do not include any personal identifiable information in your responses.   Data handling and confidentiality ·  We will have no way of connecting your survey responses to your identity. ·  We will not identify you by name in any report from this research.  ·  With your consent, your data will be shared among members of the research team. All of your answers will be treated confidentially by the research team and by anyone with whom the data is shared.   What will happen to the results of the study? The results of the study will be summarized in a report that will be presented at national and international conferences, and will be published in an academic journal.   Who should I contact for further information? If you have any questions or require more information about this study, please contact us using the following contact details: Janice Palaganas at jpalaganas@mgh.harvard.edu Julia Caton at jcaton@stanford.edu   Thank you for participating in this research.

Q61 Core Research Member    *COI* Janice Palaganas  *Secr of* Soc Sim Hc, Ctr Med Sim, Inst Ip Innov Gabe Reedy  Ed in Ch Adv Sc Clement Buleon  Ctr Med Sim, Board Mem Soc Fr Sim(SOFRASIMS) Julia Caton None to disclose Michael Buyck None to disclose Susan Eller None to disclose Suzie Kardong-Edgren *Pres El* Int Nurs Asso Cl Sim Lrng, Ctr Med Sim Juli Maxworthy *Pres El* *of* Soc Sim Hc Yoon Soo Park None to disclose
Barbara Walsh None to disclose

**Distance Simulation Offered by Your Simulation Program**

Did your simulation center conduct distance simulation activities prior to the COVID-19 pandemic?

- Yes
- No
- Not Sure

Does your simulation center currently conduct distance simulation activities?

- Yes
- No
- Not Sure

Skip To: End of Block If Does your simulation center currently conduct distance simulation activities? = No

What percentage of your simulation center’s sessions were distance simulation during the following time periods in 2020?

|  | March-May | | | | June-August | | | | September-November | | | |
| --- | --- | --- | --- | --- | --- | --- | --- | --- | --- | --- | --- | --- |
|  | Less than 50% | About 50% | More than 50% | We did not conduct simulation during this time | Less than 50% | About 50% | More than 50% | We did not conduct simulation during this time | Less than 50% | About 50% | More than 50% | We did not conduct simulation during this time |
| Distance/remote simulation |  |  |  |  |  |  |  |  |  |  |  |  |
| Hybrid remote/in-person simulation |  |  |  |  |  |  |  |  |  |  |  |  |
| In-person simulation |  |  |  |  |  |  |  |  |  |  |  |  |

Which of these factors, if any, allowed for the development of distance simulation at your simulation center? (Select all that apply)

- Access to technology to develop distance simulation
- Access to personnel able to develop distance simulation
- Interest of learners in participating in distance simulation
- Interest of simulation instructors in teaching distance simulation
- Support from the Organization (Organizational Leaders/Resources)
- Support from the Program (Simulation Program Resources)
- Other ________________________________________________
- Not sure

**Drivers for Distance Simulation**

How important were each of the following reasons for developing distance simulation at your simulation center during the COVID-19 pandemic?

|  | Not at all important | Slightly important | Moderately important | Quite Important | Extremely important | Not sure |
| --- | --- | --- | --- | --- | --- | --- |
| Request of our simulation center’s governing or accrediting body |  |  |  |  |  |  |
| Request of our simulation center’s leadership |  |  |  |  |  |  |
| Request of the participants at our simulation center |  |  |  |  |  |  |
| Request of the instructors at our simulation center |  |  |  |  |  |  |

Were there any other major reasons that your simulation center introduced or expanded distance simulation during the COVID-19 pandemic?

________________________________________________________________

How important were each of the following objectives in the transition to distance simulation at your simulation center during the COVID-19 pandemic?

|  | Not at all important | Slightly important | Moderately important | Quite important | Extremely important | Not sure |
| --- | --- | --- | --- | --- | --- | --- |
| Continuing existing simulation offerings |  |  |  |  |  |  |
| Development of new sessions related to the COVID-19 pandemic |  |  |  |  |  |  |
| Development of new sessions NOT related to the COVID-19 pandemic |  |  |  |  |  |  |

Were there any other major objectives that your simulation center was trying to achieve with the introduction or expansion distance simulation during the COVID-19 pandemic?

________________________________________________________________

What is the long-term plan for distance simulation at your simulation center?

- Plan to transition back to exclusively in-person simulation
- Plan to maintain distance simulation for some activities and transition back to in-person simulation for some activities
- Plan to transition exclusively to distance simulation
- Not sure

**Types of Distance Simulation**

What types of simulation sessions are conducted using distance simulation at your center? (Select all that apply)

- Resuscitation training, such as basic or advanced life support training (e.g. American Heart Association or other certification)
- Continuing education
- Equipment Training
- Just-in-time training
- Orientation and Competency Training
- OSCEs
- Research
- Task Training
- Team Training
- Other ________________________________________________
- Not sure

Do **educators**in your simulation center undergo any specific training for developing distance simulation sessions?

- Yes
- No
- Not sure

Do **educators** in your simulation center undergo any specific training prior to teaching in distance simulation sessions?

- Yes
- No
- Not sure

Display This Question:

If Do educators in your simulation center undergo any specific training prior to teaching in distanc... = Yes

What topics did this educator training cover? (Select all that apply)

- Teaching and learning considerations for distance education
- Technical skills needed for distance education
- Application or online platform training (If so, which applications?) ________________________________________________
- Other ________________________________________________
- Not sure

Do **simulation technicians** in your simulation center undergo any specific training for developing distance simulation sessions?

- Yes
- No
- Not sure

Display This Question:

If Do simulation technicians in your simulation center undergo any specific training for developing... = Yes

What topics does this simulation technician training cover? (Select all that apply)

- Application or online platform training (If so, which applications?) ________________________________________________
- Teaching and learning considerations for distance education
- Technical skills needed for distance education
- Other ________________________________________________
- Not sure

How are instructors at your simulation center scheduled to teach in distance simulation sessions?

- Instructors volunteer
- Instructors are chosen by simulation center leadership
- Some instructors volunteer and some instructors are chosen by simulation center leadership
- Other ________________________________________________
- Not sure

What is your impression of how satisfied each of the following groups is with distance simulation at your simulation center **currently** (September 2020 - Present)?

|  | Not at all satisfied | Slightly satisfied | Moderately satisfied | Quite satisfied | Extremely satisfied | Not applicable | Not sure |
| --- | --- | --- | --- | --- | --- | --- | --- |
| Simulation program educators |  |  |  |  |  |  |  |
| Educators who collaborate with the simulation program |  |  |  |  |  |  |  |
| Learners |  |  |  |  |  |  |  |
| Simulation technicians/Technical Specialists |  |  |  |  |  |  |  |
| Standardized patients |  |  |  |  |  |  |  |
| Simulation center staff |  |  |  |  |  |  |  |
| Instructional designers |  |  |  |  |  |  |  |
| Simulation program leaders |  |  |  |  |  |  |  |
| Organizational leaders |  |  |  |  |  |  |  |

**Methods and Modalities of Distance Simulation**

To what extent are the following simulation modalities conducted as distance simulation at your simulation center?

|  | Completely in-person | Mostly in-person, sometimes distance | About half in-person and half distance | Mostly distance, sometimes in-person | Completely distance | This modality is not used at our simulation center | Not sure |
| --- | --- | --- | --- | --- | --- | --- | --- |
| Procedural simulation |  |  |  |  |  |  |  |
| Mannequin-based simulation |  |  |  |  |  |  |  |
| Standardized patient simulation |  |  |  |  |  |  |  |
| Commercial screen-based or software simulation |  |  |  |  |  |  |  |
| Virtual reality simulation |  |  |  |  |  |  |  |

**Distance simulation process**

Did your center need to invest in any of the following resources to introduce or expand your use of distance simulation during the COVID-19 pandemic? (Select all that apply)

- Additional personnel
- Computer equipment
- Pedagogical resources
- Simulation equipment
- Video equipment
- Other ________________________________________________
- Not sure

When compared with in-person simulation, how easy or challenging is it to carry out the following aspects of distance simulation?

|  | Much more challenging than in-person simulation | Somewhat more challenging than in-person simulation | Similar to in-person simulation | Somewhat easier than in-person simulation | Much easier than in-person simulation | Not sure/no opinion |
| --- | --- | --- | --- | --- | --- | --- |
| Development of distance simulation sessions |  |  |  |  |  |  |
| Teaching in distance simulations |  |  |  |  |  |  |
| Faculty engagement in distance simulation sessions |  |  |  |  |  |  |
| Learner engagement in distance simulation sessions |  |  |  |  |  |  |
| Ability to achieve learning objectives in distance simulation sessions |  |  |  |  |  |  |

When compared with in-person simulation, how easy or challenging is it to effectively carry out each of the following simulation practices?

|  | Much more challenging than in-person simulation | Somewhat more challenging than in-person simulation | Similar to in-person simulation | Somewhat easier than in-person simulation | Much easier than in-person simulation | Not sure/no opinion |
| --- | --- | --- | --- | --- | --- | --- |
| Confidentiality |  |  |  |  |  |  |
| Pre-briefing |  |  |  |  |  |  |
| Simulation |  |  |  |  |  |  |
| Debriefing |  |  |  |  |  |  |

**Locations of Faculty and Participants**

Currently, where are the following participants located for the majority of **procedural simulation** at your simulation center?

|  | In-person in the center | In-person in the center, but separated from other participants | Online synchronously with interaction (audiovisual or chat) | Online synchronously without interaction (observation only) | Online asynchronously | Not present | Not sure | This modality is not used in our program |
| --- | --- | --- | --- | --- | --- | --- | --- | --- |
| Active learners |  |  |  |  |  |  |  |  |
| Observing learners |  |  |  |  |  |  |  |  |
| Instructors |  |  |  |  |  |  |  |  |
| Simulation techs |  |  |  |  |  |  |  |  |
| Administrative staff |  |  |  |  |  |  |  |  |

Currently, where are the following participants located for the majority of **clinical event-based or team-based simulation** at your simulation center?

|  | In-person in the center | In-person in the center, but separated from other participants | Online synchronously with interaction (audiovisual or chat) | Online synchronously without interaction (observation only) | Online asynchronously | Not present | Not sure | This modality is not used in our program |
| --- | --- | --- | --- | --- | --- | --- | --- | --- |
| Active learners |  |  |  |  |  |  |  |  |
| Observing learners |  |  |  |  |  |  |  |  |
| Instructors |  |  |  |  |  |  |  |  |
| Simulation techs |  |  |  |  |  |  |  |  |
| Administrative staff |  |  |  |  |  |  |  |  |

Currently, where are the following participants located for the majority of **simulation with simulated patients (SPs)** at your simulation center?

|  | In-person in the center | In-person in the center, but separated from other participants | Online synchronously with interaction (audiovisual or chat) | Online synchronously without interaction (observation only) | Online asynchronously | Not present | Not sure | This modality is not used in our program |
| --- | --- | --- | --- | --- | --- | --- | --- | --- |
| Active learners |  |  |  |  |  |  |  |  |
| Observing learners |  |  |  |  |  |  |  |  |
| Instructors |  |  |  |  |  |  |  |  |
| Simulation techs |  |  |  |  |  |  |  |  |
| Administrative staff |  |  |  |  |  |  |  |  |
| Simulated/standardized patients |  |  |  |  |  |  |  |  |

End of Block

Start of Block: demographics

**Demographic Questions (All questions in this section are optional )**

| 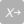 |
| --- |

In which country is your simulation center located? (Optional)

▼ Afghanistan ... Zimbabwe

Display This Question:

If List of Countries = United States of America

In what state is your simulation center located? (Optional)

▼ Alabama ... I do not reside in the United States

What is the name of your simulation center? (Optional)

________________________________________________________________

What is the name of your institution? (Optional)

________________________________________________________________

What is your role at your simulation center? (select all that apply) (Optional)

- Simulation educator
- Industry developer/sales
- Simulation operations specialist/technician
- Simulation program director/administrator
- Simulation researcher
- Standardized patient program director/administrator
- Other

How much of your time is formally dedicated to a simulation program? (Optional)

- Less than 50%
- 50-75%
- More than 75%

What type of organization your simulation center program provides activities for? (Select all that apply) (Optional)

- College, university, or high-education academic institution
- Community
- Hospital
- Industry
- Institutional (college, university, or higher-education academic institution + academic medical center)
- Private
- Other ________________________________________________
- Not sure

What professions does your simulation center support? (Select all that apply) (Optional)

- Advanced practice providers (e.g. nurse practitioners, physician assistants)
- Allied health providers
- Child life specialists
- Dentists or dental students
- Nurses or nursing students
- Occupational therapists
- Paramedics, prehospital, EMS
- Pharmacists
- Physical therapists
- Physicians or medical students
- Respiratory therapists
- Social workers
- Other ________________________________________________
- Not sure

What is the most appropriate description for the physical program type of your simulation program? (Optional)

- Two or fewer full-time employees dedicated to the program
- Three to six full-time employees dedicated to the program
- Seven or more full-time employees dedicated to the program
- Not sure

What is the most appropriate description of the utilization type for your simulation program? (Optional)

- 2,500 to 5,000 learner contact hours per year
- 5,001 to 10,000 learner contact hours per year
- 10,001 or more learner contact hours per year
- Not sure

Skip To: End of Survey If What is the most appropriate description of the utilization type for your simulation program? (Op... , 2,500 to 5,000 learner contact hours per year Is Displayed

End of Block: demographics

Start of Block

Which of these factors, if any, are reasons that your center is not currently conducting distance simulation? (select all that apply)

- Continued in-person training
- Decision to pause simulation training altogether
- Lack of access to technology to develop distance simulation
- Lack of access to personnel able to develop distance simulation
- Lack of interest of learners in participating in distance simulation
- Lack of interest of simulation instructors in teaching distance simulation
- Lack of organizational support (Organizational Leaders/Resources)
- Lack of programmatic support (Simulation Program Resources)
- Other ________________________________________________
- Not Sure

Q44 Do you have any other thoughts that you would like to share? (Optional)

________________________________________________________________

Skip To: End of Block If Condition: Do you have any other thoug... Is Displayed. Skip To: End of Block.

End of Block

Start of Block

Demographic Questions (All questions in this section are optional))

| 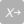 |
| --- |

In which country is your simulation center located? (Optional)

▼ Afghanistan ... Zimbabwe

Display This Question:

If List of Countries = United States of America

In which state is your simulation center located? (Optional)

▼ Alabama ... I do not reside in the United States

What is the name of your institution? (Optional)

________________________________________________________________

What is your simulation center's name? (Optional)

________________________________________________________________

What is your role at your simulation center? (Select all that apply) (Optional)

- Simulation educator
- Industry developer/sales
- Simulation operations specialist/technician
- Simulation program director/administrator
- Simulation researcher
- Standardized patient program director/administrator
- Other

How much of your time is formally dedicated to a simulation program? (Optional)

- Less than 50%
- 50-75%
- More than 75%

What type of organization your simulation center program provides activities for? (Select all that apply) (Optional)

- Hospital
- College, university, or higher education academic institution
- Institutional (college/university/higher education academic institution + academic medical center)
- Community
- Industry
- Private
- Other ________________________________________________
- Not sure

What professions does your simulation center support? (Select all that apply) (Optional)

- Advanced practice providers (e.g. nurse practitioners, physician assistants)
- Allied health providers
- Child life specialists
- Dentists or dental students
- Nurses or nursing students
- Occupational therapists
- Paramedics, prehospital, EMS
- Pharmacists
- Physical therapists
- Physicians or medical students
- Respiratory therapists
- Social workers
- Other ________________________________________________
- Not sure

What is the most appropriate description for the physical program type of your simulation program? (Optional)

- Two of fewer full-time employees dedicated to the program
- Three to six full-time employees dedicated to the program
- Seven or more full-time employees dedicated to the program
- Not sure

What is the most appropriate description of the utilization type for your simulation program? (Optional)

- 2,500 to 5,000 Learner contact hours per year
- 5,001 to 10,000 Learner contact hours per year
- 10,001 Or more learner contact hours per year
- Not sure

Do you have any other thoughts that you would like to share? (Optional)

________________________________________________________________
